# Assessing the reliability of camera-based identification, activity monitoring, and location in housing systems on dairy farms

**DOI:** 10.3168/jdsc.2025-0886

**Published:** 2026-01-30

**Authors:** Anne-Cécile Toulemonde, Aurélien Madouasse, Yannick Le Cozler, Raphaël Guatteo

**Affiliations:** 1 Oniris, INRAE, BIOEPAR, 44300 Nantes, France; 2 PEGASE, INRAE, Institut Agro, 16 Le Clos, 35590 Saint Gilles, France

## Abstract

Video-based livestock monitoring offers a noninvasive, cost-effective, and scalable alternative to direct human monitoring, but also to commonly used collar or ear tag devices on farms. It enables simultaneous real-time observation of multiple animals while avoiding stress and injuries from physical devices. However, single-camera systems face challenges such as blind spots and limited individual tracking, especially in barns lacking corridor layouts. These limitations can be overcome using multi-camera, multi-cow tracking (MCMCT) systems that integrate deep learning and statistical techniques to enable continuous detection, identification, activity classification, and zone location of animals in the barn, under commercial conditions. This environment is characterized by high stocking density (in m^2^ per cow), occlusions, and variable lighting. In this study, a commercial MCMCT system was tested over 31 d (May 2025) on 3 Holstein dairy farms in western France. Herd size ranged from 70 to 250 lactating cows and used automatic milking systems (AMS), which allowed identification of all animals when milked. Individual detection performance of this MCMCT system was then validated compared with official AMS records. A dedicated hybrid confusion matrix framework was developed to jointly assess detection and identification errors in the sequential process, allowing precise calculation of recall, precision, and F1-scores at both stages. Overall, this MCMCT system achieved over 90% detection recall and 87% to 93% precision, successfully detecting continuously more than 9 out of 10 cows daily. Identification was more challenging, with recall varying from 69% to 78% and precision above 83%, resulting in F1-scores of 79% to 82%. The performance of detection varied significantly between day and night in 2 out of 3 farms (H1 and H2), with recall rates dropping to 76% at night and exceeding 94% during peak daylight, underscoring the impact of lighting and activity patterns. Activity classification and zone location were robust, with F1-scores exceeding 87%, demonstrating the system's capacity to provide practical insights for herd management such as monitoring individual behaviors, identifying high-density zones around resources, and supporting daily management decisions. This work confirms the system's practical viability as a scalable, noninvasive monitoring solution effective under commercial farm complexities such as crowding, occlusion, and lighting variability. The integration of day–night performance analysis and the hybrid confusion matrix provide a rigorous and transparent framework for assessing system reliability, critical for deploying precision livestock farming technologies. Identification performance decreased under overcrowded conditions. Overcrowding is defined here as a surface area of less than 9 m^2^ per cow or less than one cubicle per cow, as recommended by the EFSA Panel on Animal Health and Animal Welfare in 2023. The system demonstrates significant potential to support and enhance herd management, early disease detection, and animal welfare monitoring.

## Highlights

• Cow detection: recall 90.6%-99.6%, reliably detecting >9/10 cows daily, robust across day despite lighting and overcrowding.
• Cow identification: recall 69.1%-78.1%, precision 83.2%-91.3%, correctly identifying approximatively 7-8/10 cows.
• Activity and location: F1-scores 87.5%-92.4% for correctly identified cows.

## Graphical Abstract

**Figure:**
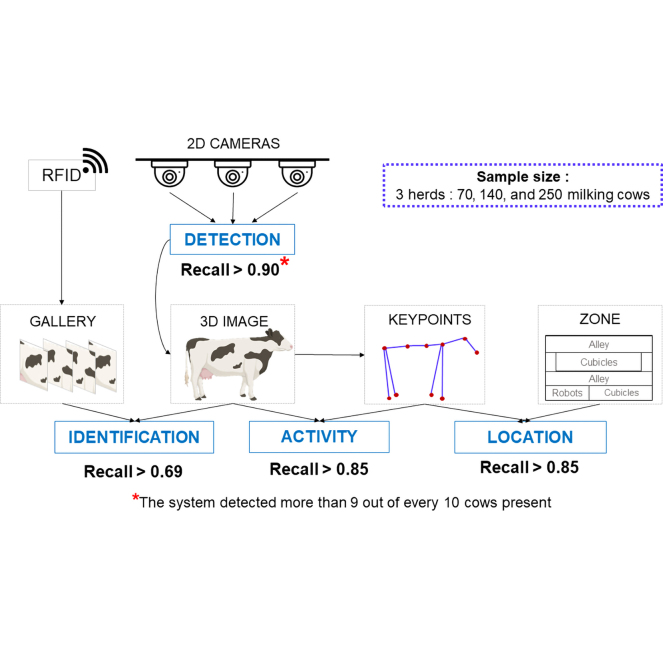
**Summary:** A multi-camera, multi-cow tracking system was evaluated on 3 commercial dairy farms in western France, monitoring 70, 140, and 250 lactating Holstein cows in May 2025. The system reliably detected over 9 out of 10 cows daily, with recall ranging from 90.6% to 99.6%. Detection showed daily variation across herds, but overall system performance remained robust, with hourly detection rates above 75% and most values exceeding 85%, despite lighting and overcrowding challenges. Cow identification achieved recall of 69.1% to 78.1% and precision of 83.2% to 91.3%, correctly identifying approximately 7 to 8 out of 10 cows. Activity classification and zone location reached F1-scores of 87.5% to 92.4% for well-identified cows. Optimizing performance requires reducing overcrowding, maintaining cameras, and ensuring cow cleanliness. Overall, the system demonstrates practical utility for continuous, noninvasive herd monitoring, though further refinement is needed before replacing human expertise. 2D = 2-dimensional; 3D = 3-dimensional; RFID = radio-frequency identification.

Over the past 20 years, the number of dairy farms in Europe has declined by 38%, whereas the average herd size has increased from 32 to 49 cows and milk yield per cow has increased by 37% (European Commission, 2021). These changes have been accompanied by a shrinking and aging workforce, along with increasing specialization (Augere-Granier and Vinci, 2018). In this context, precision livestock farming plays a central role in managing herds efficiently and sustainably, particularly in the case of health management. Indeed, monitoring movement patterns provides valuable insights into changes in time budgets, activity levels, or social behaviors (or a combination of these), which can be leveraged to detect health disorders such as lameness (Russello et al., 2024). Information from monitoring patterns can also be used to support effective reproductive management such as heat detection and calving (Benaissa et al., 2020). This requires the ability to efficiently detect and track individual cows' behavior over time and space. In contrast to sensors attached to individual cows, such as collars, ear tags, or leg-mounted accelerometers (Norton and Berckmans, 2017), video-based systems offer a noninvasive, cost-effective, multifunctional, sustainable, and scalable alternative for indoor livestock monitoring. Compared with visual appraisal, they enable real-time observation of multiple animals simultaneously, in both research and farming settings (Faverdin et al., 2020). Additionally, these systems avoid the stress and possible injuries caused by installation and maintenance (Norton et al., 2019), while enabling the integration of multiple sources of information. Single-camera systems are available and used on farms (Swartz et al., 2025); however, such setups often suffer from blind spots, limiting their ability to capture cow movements continuously. In addition, they struggle to accurately identify individual cows because partial views and varying angles often lead to missing or overlapping images (Yamamoto et al., 2025). Furthermore, these systems are limited to narrow barns with few cows, equipped with corridors suitable for single-camera or single-spot-based systems, and when available, this limits the ability to track animals throughout the day. As a result, multi-camera, multi-cow tracking (**MCMCT**) systems have emerged as a promising approach to provide continuous, automated, and spatially detailed individual-level information (Xu et al., 2025). These systems cover the entire field of view, capturing animal information from all positions and angles. This comprehensive coverage makes them more flexible and adaptable than single-camera systems, regardless of barn configuration. The MCMCT systems have shown promising results at the experimental scale in loose housing or bedded pack environments, where visual occlusions are minimal and cow density is lower or better distributed than in commercial settings (Xu et al., 2025; Yamamoto et al., 2025). A commercial MCMCT solution for dairy cattle in France (AiHerd) combines deep learning and statistical methods to continuously and spatially track animals and their behaviors in operational commercial freestall barns. Therefore, the objectives of the present study were to evaluate this system's ability to correctly detect and identify cows, as well as to determine their activity (eating, drinking, lying, standing, walking, waiting) and localize them within freestall barns. The system was tested under commercial farm constraints, including high-density housing, occlusions, and lighting variability, while also addressing the practical feasibility of its implementation on farm.

The study was conducted in 3 Holstein dairy farms (**H1**, **H2**, **H3**) in western France. Selected farms represented a range of herd sizes and some typical layouts of France's intensive dairy production system. Holsteins are easily recognizable by their distinctive black-and-white coat pattern, making them particularly well-suited for visual identification. Briefly, H1 had 70 lactating cows and a barn area of approximately 625 m^2^, equipped with one AMS; H2 had 140 lactating cows and a 1,225 m^2^ barn with 2 AMSs; H3 had 250 lactating cows and an 1,820 m^2^ barn with 5 AMS. This corresponded to stocking densities of 8.9, 8.8, and 7.3 m^2^ per cow, respectively. All animals were housed in freestall barns with 66 (H1), 132 (H2), and 218 (H3) cubicles, with 2, 5, and 8 water troughs, respectively. The average daily milk production per cow was 35, 35, and 31 kg/d in H1, H2, and H3, respectively. The cows were fed a TMR once daily in the morning between 0700 and 0900 h across all farms, with an automatic feed pushing system in H2 and H3. As this study was conducted on commercial farms without any direct intervention on animals, it did not require approval from an ethics committee under French regulations. In addition, the farmers signed an informed consent at the beginning of the study.

Cows had no outdoor access and were monitored using the MCMCT system. This system combined cameras and stationary radio-frequency identification (**RFID**) readers. Cameras (Uniview IPC815SB-ADF14K-I0) had a resolution of 5 megapixels. They were equipped with a 1/2.8” (≈6.46 mm diagonal) Complementary Metal-Oxide-Semiconductor (CMOS) sensor and a fixed 1.4-mm fisheye lens allowing 360° panoramic views and capture videos at 2,592 × 1,944 pixels and 30 frames per second. Infrared vision at night enables monitoring up to 10 m, with a minimum light sensitivity of 0.01 lx. Cameras were connected via RJ45 Ethernet cables to a local server installed on-site. The number of cameras installed per barn was 3, 8, and 14 in H1, H2, and H3, respectively, corresponding to camera-to-cow ratios of approximately 1:23.3, 1:17.5, and 1:17.9. All were placed under the barn ridge, spaced 14.5 m apart in H1, 13.4 m in H2, and 13.8 m in H3, at heights of 4.9, 5.7, and 6.0 m, respectively. In addition, stationary RFID readers were used to detect cow identity. Depending on the farm layout, they were installed either in the AMS (H1 and H2) or at entry-exit waiting area (H3). The RFID sensors inform the deep learning algorithms in real time: images of cows are captured as they pass in front of the RFID reader and within a few meters afterward. To avoid misidentifying cows, individual trajectories were tracked fully automatically after passing the reader. Each observed trajectory was compared with a pool of reference trajectories obtained during the calibration phase using the Fréchet distance, thereby ensuring correct association between the RFID tag and the corresponding images.

The MCMCT pipeline is composed of a structured sequence of steps, starting with cow detection, followed by 3-dimensional reconstruction and tracking to enable individual identification. Afterward, a data aggregation integrated activity classification (eating, drinking, lying, standing, walking, waiting) and zone location (alley, drinking trough, feeding trough, cubicle, AMS, AMS waiting area) in the barn, as the position of the camera is precisely known. In this study, waiting activity was defined as standing in designated areas before entering the milking system (AMS waiting area).

All the lactating cows passing through the AMS and identified thanks to their RFID ear tag were included in this study. Collected data contained the following fields: cow ID, timestamp (date and time when the data were recorded), activity classification, and barn zone location. These data were divided into 2 parts: one to evaluate detection performance and the other to assess individual identification, activity classification, and zone location within the barns.

To assess detection performance, data were collected from May 1 to June 1, 2025, providing 31 d of observations to capture daily variability. Two datasets were used for this evaluation: (1) the AMS dataset, which recorded the date and time of each cow's visit to the AMS, and (2) the MCMCT dataset, which contained the list of detected cows at each time point. Each day, the list of cows visiting the AMS at least once between 0000 and 2359 h was compared with the MCMCT detection list, yielding 31 daily data points per farm.

In addition, detection stability was assessed. Detection stability, defined as the consistency of the MCMCT system's cow detection performance throughout the day, was quantified by the proportion of cows detected in each hourly interval. The number of cows detected simultaneously was recorded every 5 min, resulting in 288 data points per day per farm and 8,928 data points per farm over the study period. These values were then summed on an hourly basis, and the performances of the MCMCT system regarding cow identification, activity classification, and location were evaluated by comparing the system output with visual observations.

To determine the required sample size for visual observations, the most error-prone step in the pipeline first had to be identified. Individual cow identification is a critical step in the pipeline, with each animal having approximately a 1/*n* probability of correct identification, where *n* represents the total number of cows. Identification was continuously challenged in the MCMCT pipeline to ensure that 2 cows could not have been assigned the same identification number simultaneously. Activity and location involved few categories, resulting in fewer errors in attribution than individual identification. For this reason, the sample size was calculated based on the identification objective.

Accordingly, the number of visual observations to collect was calculated, using a population proportion sample size calculator (Dhand and Khatkar, 2014). As no prior performance data were available for the same device under similar conditions, the calculation used conservative assumptions to ensure that the minimum required sample size would be sufficient in practice. Consequently, the expected proportion of correctly identified images was set at 50%, with a 5% absolute margin of error and a 95% confidence level. The sample size was calculated to meet the expected performance and adjusted for finite population. This resulted in minimum sample sizes of 60, 106, and 140 images per farm for identification, activity classification, and zone location, respectively. These minimums refer to the statistical requirement per measurement type, but in practice, all visible cows were included, yielding final sample sizes larger than the minimums without selectively targeting individual animals. This approach also ensured full activity variability and spatial coverage across all barn zones. Thus, the final sample sizes exceeded the calculated minimums of H1 = 80, H2 = 151, and H3 = 187. Data were collected on farms on May 25 (H1 and H2) and May 26 (H3), 2025. Identification, activity classification, and zone location were assessed through direct observations on the farm by a single observer considered as the gold standard to ensure consistency across farms and measurement types. The MCMCT operated in real time, updating approximately every minute. The observer walked through the barn and, for each cow encountered, compared its actual identity, activity, and location with the current MCMCT output. The observer validated the results every 1 to 3 min (depending on the time needed to locate the next cow) by refreshing the MCMCT display, visually checking both ear tags, the cow's activity, and its barn area, recording the corresponding output, and then proceeding to the next animal to maintain temporal alignment.

In the pipeline used, a cow must first be present and be detected (detection) in the observation area before being assigned an identity based on coat pattern (identification). A hybrid confusion matrix that merged the detection and identification confusion matrix into a single one was built to reflect this sequential dependency (Figure 1). For detection (**D**), a case was considered a true positive (**TP-D**) when a cow was present in the herd (as confirmed by the daily identity records from the AMS) and correctly detected by the system. A false negative (**FN-D**) occurred when a cow was truly present in the herd but not detected by the system. Conversely, a false positive (**FP-D**) corresponded to a detection made for a cow that was absent in the herd (i.e., not listed in the daily identity records). True negatives (**TN-D**), defined as the absence of both cows and detection, did not exist in our study because all animals in the camera field belonged to the herd and were traceable.

**Figure 1:**
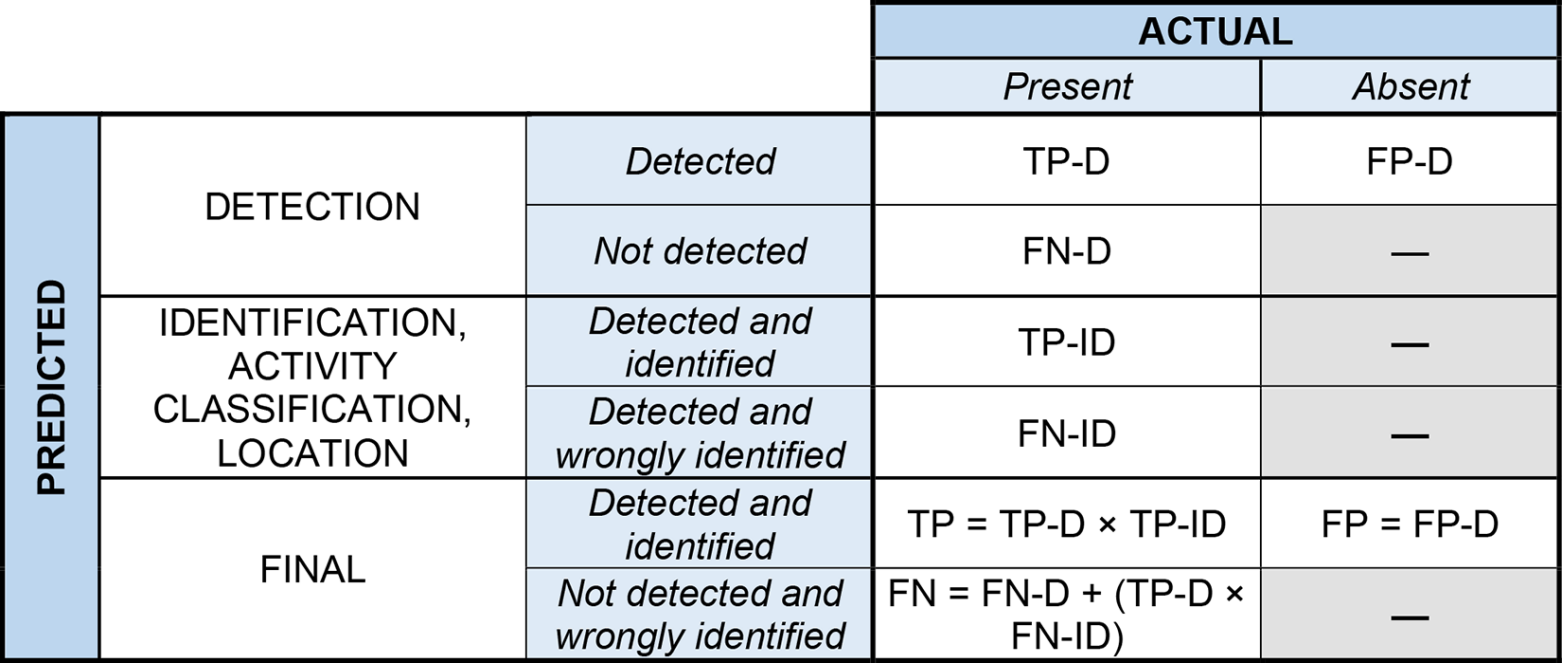
Hybrid confusion matrix framework for performance assessment of detection, identification, activity classification, and location. TP-D = true positive detection; FN-D = false negative detection; FP-D = false positive detection; TP-ID = true positive identification; FN-ID = false negative identification; TP = cow correctly detected and identified; FN = cow missed or misidentified; FP = cow present but not detected.

To assess the stability of detection throughout the day, the number of cows simultaneously detected was recorded every 5 min. The data were first averaged within each hour to obtain hourly mean counts, which were then converted into proportions of cows detected (number of cows passing in the AMS). A linear mixed-effects model was fitted on each farm to evaluate whether these hourly proportions varied significantly over the 24 h. The hour of the day was included as a fixed effect. Day was included as a random effect to account for repeated measurements within days. In case of significant effect of hour, post hoc pairwise comparisons were performed. Estimated marginal means with Tukey adjustment identified specific hours with statistically different detection rates (*P* < 0.01). All analyses were performed using RStudio (4.5.1), with the *lme4* and *emmeans* packages.

Once detection was performed, it was possible to evaluate the performance of the system for identification (**ID**), activity classification, and location based on a similar confusion matrix structure than previously described. Thus, applying this method to identification, a case was considered as a true positive (**TP-ID**) when a cow was detected by the system, and her identity assigned by the system matched with her official ear tag number. A false negative (**FN-ID**) occurred when a detected cow was either misidentified or not identified at all by the system. Since cows must first be detected before being identified, false positives (**FP-ID**) and true negatives (**TN-ID**) could not be calculated, as they could not exist. The final confusion matrix for both cow detection and identification is displayed in Figure 1. A final true positive (**TP**) occurred when a cow was present in the herd, correctly detected, and correctly identified. False negative (**FN**) referred to a cow present in the herd, correctly detected, but misidentified or not identified, or a cow that was present but missed by the detection system. False positive (**FP**) occurred when a cow was present but not detected, and therefore could not be identified. This hybrid confusion matrix enabled the computation of performance evaluation metrics: recall, precision, and F1-score. Recall measures how many of the actual cows present were detected by the system. Precision measures how many of the detected cows were truly present. F1-score combines precision and recall to measure how well the system detects cows while minimizing missed detections and false alarms. The respective equations used to calculate these parameters are detailed as follows:

$$r\mathrm{ecall}=\;\frac{\mathrm{TP}}{\left(\mathrm{TP}+\mathrm{FN}\right)},$$

$$\mathrm{precision}=\frac{\mathrm{TP}}{\left(\mathrm{TP}+\mathrm{FP}\right)},$$

$$F1-\mathrm{score}=2\times \frac{\mathrm{precision}\times \mathrm{recall}}{\mathrm{precision}+\mathrm{recall}\;}.$$

Detection performance was high on all farms, with a recall of 90.6% in H1, 92.8% in H2, and 99.6% in H3, and a precision of 93.2%, 87.9%, and 86.7%, respectively. This means the system successfully detected more than 9 out of every 10 cows present throughout the day (recall), and that around 8 to 9 out of every 10 cows identified by the system were indeed present (precision). These performances are consistent with the precision of 78% and recall of 88% reported by Das et al. (2025) in a commercial freestall Holstein farm (107 cows) equipped with 7 cameras. The high detection recall in the present study provided a reliable foundation for subsequent steps, including activity classification and individual identification. Results showed significant daily variation across all herds (Figure 2). For example, for H1 herd, detection ranged from 75.6% at 0500 h (SE = 1.03%, *P* < 0.001) to 94.0% at 0800 h (SE = 1.03%, *P* < 0.001). Post hoc comparisons (Tukey-adjusted) confirmed that detection was lower during night-time hours (0300–0500 h) compared with the daytime period (0800–2000 h; *P* < 0.001). In H2, detection levels were generally higher, ranging from 91.5% at 0800 h (SE = 0.65%) to a peak of 104.8% at 2100 h (SE = 0.65%). Similarly to H1, the model showed hour effects (*P* < 0.001). These higher daytime values are likely due to overdetection caused by lighting conditions and animal density. In contrast, H3 exhibited the most stable detection performance, with values ranging from 80.5% at 0400 h (SE = 1.55%) to 88.8% at 1600 h (SE = 1.55%; Figure 2). Although the variation was less pronounced, differences between some night and day periods remained statistically significant. These day–night differences might be explained by the lighting strategy. Indeed, H1 and H2 kept neon lights on overnight between cubicles, whereas H3 used spaced-out LED lamps. Moreover, dairy cows spend more time lying in cubicles at night (Hut et al., 2022), which aligns with these lighting conditions. Altogether, these factors likely explain the higher night-time detection rates observed in H1 and H2, as well as the more stable performance in H3. Furthermore, the stability of H3's detection performance over the day can be attributed to a balance between lower detection rates in the AMS waiting area, which is less well covered by cameras and prone to local overcrowding during the day and reduced lighting conditions at night. This variation in detection is particularly important for key events such as calving, which often occur at night and require reliable, continuous monitoring. Overall, day–night detection gaps remained within the range of 10%, consistent with Das et al. (2025), which indicates that this approach is suitable for an optimized 24-h monitoring performance approach that can be used on farms.

**Figure 2:**
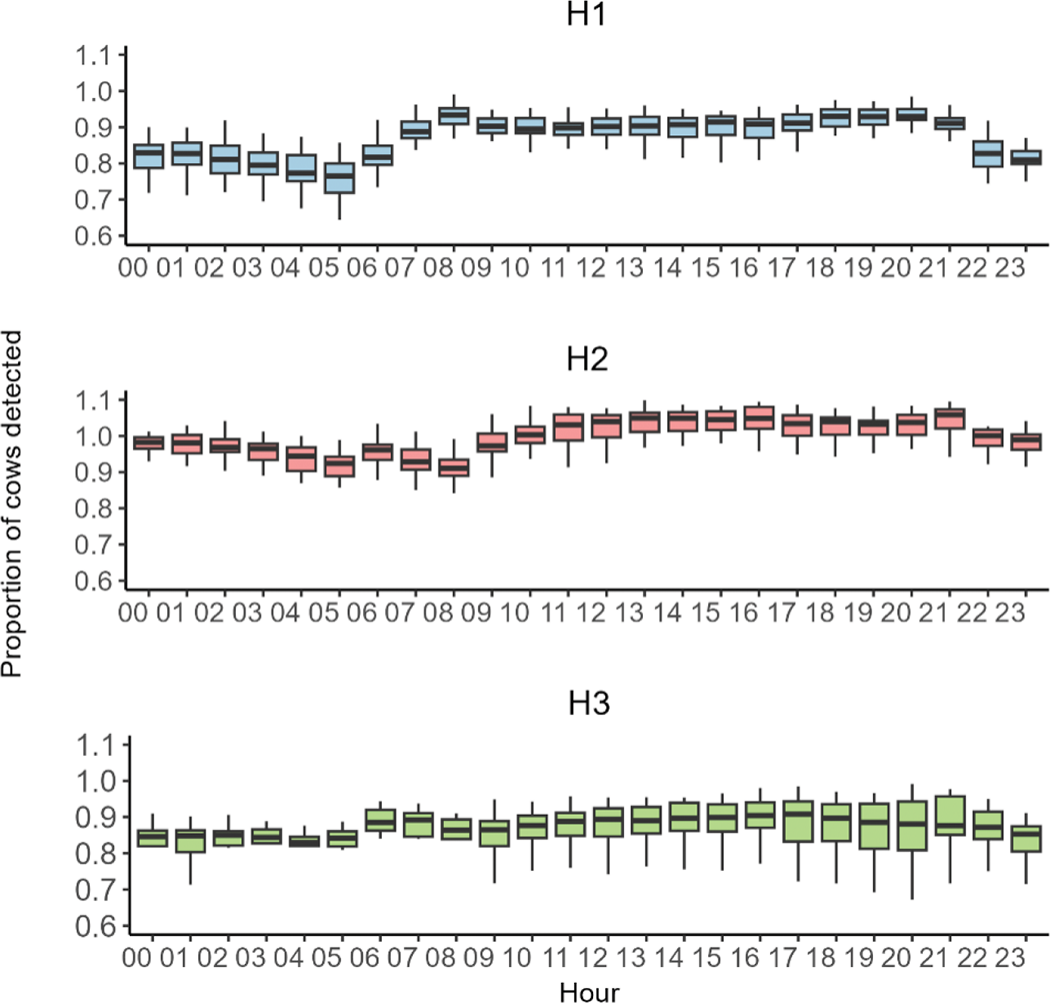
Distribution of proportion of cows detected per hour per herd (H1, 70 cows; H2, 140 cows; and H3, 250 cows). Each box displays the distribution through 5 summary values: minimum (lower end of vertical line), first quartile (bottom of the box), median (line inside box), third quartile (top of the box), and maximum (upper end of vertical line).

Identification recall ranged from 69.1% to 78.1% (Table 1), meaning the system correctly identified approximatively 7 to 8 out of every 10 cows present throughout the day. Precision values are between 83.2% and 91.3%, indicating that approximately 8 to 9 out of every 10 identifications corresponded to actual cows. In complement, F1-scores ranged from 78.6% to 81.8%.

**Table 1:** Performance of identification, activity classification, and location of cows in 3 farms (H1, 70 cows; H2, 140 cows; and H3, 250 cows)^1^

Item	TP (%)	FN (%)	FP (%)	Recall	Precision	F1-score
Identification						
H1	69.01	30.94	6.57	69.05	91.31	78.61
H2	78.05	21.95	12.76	78.05	85.95	81.77
H3	75.36	24.64	15.22	75.36	83.22	79.03
Activity classification						
H1	84.78	15.10	6.57	84.88	92.80	88.66
H2	87.86	12.13	12.76	87.86	87.33	87.48
H3	99.01	0.99	15.22	99.01	86.68	92.39
Location						
H1	84.78	15.10	6.57	84.88	92.80	88.66
H2	92.79	7.21	12.76	92.79	87.91	90.31
H3	99.01	0.99	15.22	99.01	86.68	92.39

1 TP = true positive; FN = false negative; FP = false positive.

All these results were obtained under commercial farm conditions, with 70 to 250 dairy cows, stocking densities ranging from 7.3 to 8.9 m^2^ per cow, fewer than one cubicle per cow, and camera-to-cow ratios from 1:17.5 to 1:23.3 In this study, “overcrowding” refers to the combination of high stocking density and resulting local crowding around resources, defined as conditions below the European Food Safety Authority (EFSA) recommendations of ≥9 m^2^ per cow and ≥1 cubicle per cow (EFSA Panel on Animal Health and Animal Welfare et al., 2023). This overcrowding likely contributed to challenges in individual detection and identification, especially around drinkers and in AMS waiting areas. These conditions differ from those described in control studies using multi-camera systems with diagonal views based on back and trunk pattern (Xiao et al., 2022). These authors reported better performances, with a notably higher F1-score of 97.3%, a 98.2% precision, and a recall of 96.5%. However, Xiao et al. (2022) used only 24 Holstein cows in a freestall setting, under more favorable controlled experimental conditions (15.63 m^2^/cow, 1 camera per 4 cows), far from the situation encountered in most commercial farms. Similarly, Yamamoto et al. (2025) achieved an identification F1-score of 89.2%, but in favorable experimental conditions, with diagonal angle-view in a nonobstructed compost barn and a low stocking density (13.1 m^2^/cow). In contrast, Tassinari et al. (2021) reported a lower F1-score of 62%, mainly due to limited recall (45.6%), likely due to their focus on hip-only regions, a single-camera setup placed at 3.5 m and density (8.0 m^2^/cow). All these contrasting results underscore the significant impact of environmental complexity such as freestall versus open-space housing and camera density per animal on identification performance. The effectiveness of identification also depends on animal characteristics as reported in previous studies (Das et al., 2025; Xu et al., 2025). Larger cows with distinct coat patterns are more reliably detected, especially in crowded conditions. Although most current algorithms rely on coat pattern differences, recent studies show that shape-based detection methods can successfully track unicolor breeds like Angus cattle, although these have so far been tested only in small-scale, controlled experiments (Myat Noe et al., 2023). A tentative attempt to define an optimal camera density suggests about 8 to 10 cows per camera, depending on barn layout and blind spots, to balance coverage quality and cost-effectiveness (Sourav and Peschel, 2022).

Although individual identification performance remains moderate (F1-score between 78.6% and 81.8%), this level can be considered acceptable for health monitoring applications, where activity is assessed over hours or days rather than in real time. Heat-related behaviors associated with estrus (e.g., increased activity, mounting) typically last several hours. Artificial insemination or mating usually occurs 12 to 24 h after the onset of these behaviors, giving the system sufficient time to detect and identify the events correctly. Lameness detection can rely on moment-to-moment observation of locomotion (Russello et al., 2024), but it can also rely on day-to-day variations in activity patterns (de Mol et al., 2013). Unlike estrus, lameness develops gradually, so monitoring focuses on early deviations to enable timely intervention rather than capturing the exact onset. In both cases, consistent attribution of the majority of activity data is sufficient to generate timely and actionable alerts.

Supporting this, Yamamoto et al. (2025) demonstrated that a location-based multi-camera tracking system with an ID F1-score of ∼80% and multiple object tracking accuracy of ∼90% could support reliable health and reproductive monitoring across a commercial dairy barn. These findings underscore the practical viability of consistent and reliable identification accuracy. Incorporating shape-based recognition within the current setup (Chen et al., 2021) could enhance performance in the future.

Activity classification performance was strong across farms, with recall above 84.9%, and precision ranging from 86.7% to 92.8%, resulting in F1-scores varying from 87.5% to 92.4%. Zone location performance mirrored activity classification, indicating that most of the time, the system made both activity classification and location errors for the same cow at the same time. However, this co-occurrence is not systematic and depends on the activity type and zone size, with more spatially restricted behaviors (e.g., feeding or drinking) showing only limited overlap compared with activities in larger areas (e.g., standing or lying in alleys and cubicles). To put these findings in context, they can be compared with alternative localization approaches. Animal location in barns is usually achieved using real-time location systems (**RTLS**), such as ultra-wideband– or global navigation satellite system–based navigation (Mao et al., 2023). Moreover, most RTLS-based studies emphasize accuracy as the primary performance metric, whereas few consider recall as a key measure. Chapa et al. (2021) collected data from 40 lactating cows (39 Brown Swiss and 1 Holstein Friesian) housed in a freestall barn using RTLS. They reported recall values ranging from 71.7% in alleys to 93.5% in feeding areas, with precision between 88.7% and 92.4% for these zones. More recently, Melzer et al. (2021) reported sensitivities ranging from 80% in the feeding bunk area to 99% in the cubicles, alongside precision values between 85% in walking alleys and 97% in lying cubicles, based on data collected in a 161.3-m^2^ pen housing 15 dairy cows. Despite a greater diversity of functional zones, a larger monitored area, higher cow density, and more occlusion challenges, the system we used in the present study achieved comparable or superior performance, highlighting its robustness and scalability in complex barn environments.

The hybrid evaluation framework that was developed here can be useful for possible users who are not directly familiar with the tool used, do not have the necessary resources to continuously record and process video data, or both. To be deployed in commercial farming conditions, this framework combines data routinely collected by the system with occasional human observations, considered as the gold standard. The choice of standard metrics (recall, precision, and F1-score) allows the results to be translated into concrete numbers of cows correctly detected or identified.

The present study evaluated an MCMCT system dedicated for accurate and stable cow detection, individual identification, activity classification, and location within functional zones in freestall barns across 3 commercial farms. Despite real-life challenges such as blind spots, overcrowding, and variable lighting, the system successfully detected over 9 out of every 10 cows present and identified approximatively 7 to 8 out of every 10 individual cows, achieving performance comparable to controlled experimental setups. Some cows were not consistently identified during the study period, primarily due to coat color (fully black or fully white), although commercial conditions also contributed to the misidentifications, revealing inherent limitations of the system. This tool can serve as a valuable decision-support aid for farmers in the daily management of their herd. Improving resource access (e.g., water points) to reduce overstocking, optimizing lighting strategies, ensuring camera maintenance, and selecting cows with distinctive coat patterns or ensuring cows' cleanliness to avoid slurry contamination of the coat can enhance system performance. This technology offers a promising, noninvasive tool for continuous monitoring and early detection of behavioral changes, supporting timely management decisions and herd welfare. The implementation of the system in commercial farms requires a comprehensive installation process, including an initial barn scan and environment-specific calibration. Additionally, this technology could monitor other health and welfare indicators, such as lameness, respiratory rate, heat stress, or social interactions. This would broaden its applications in commercial livestock management.
